# Porcine endometrial heat shock proteins are differentially influenced by pregnancy status, heat stress, and altrenogest supplementation during the peri-implantation period

**DOI:** 10.1093/jas/skac129

**Published:** 2022-07-01

**Authors:** Malavika K Adur, Jacob T Seibert, Matthew R Romoser, Katie L Bidne, Lance H Baumgard, Aileen F Keating, Jason W Ross

**Affiliations:** Department of Animal Science, Iowa State University, Ames, IA 50011, USA; Department of Animal Science, Iowa State University, Ames, IA 50011, USA; Department of Animal Science, Iowa State University, Ames, IA 50011, USA; Department of Animal Science, Iowa State University, Ames, IA 50011, USA; Department of Animal Science, Iowa State University, Ames, IA 50011, USA; Department of Animal Science, Iowa State University, Ames, IA 50011, USA; Department of Animal Science, Iowa State University, Ames, IA 50011, USA

**Keywords:** altrenogest, endometrium, heat shock proteins, heat stress, pigs, reproduction

## Abstract

Heat stress (**HS**) deleteriously affects multiple components of porcine reproduction and is causal to seasonal infertility. Environment-induced hyperthermia causes a HS response (**HSR**) typically characterized by increased abundance of intracellular heat shock proteins (**HSP**). Gilts exposed to HS during the peri-implantation period have compromised embryo survival, however if (or how) HS disrupts the porcine endometrium is not understood. Study objectives were to evaluate the endometrial HSP abundance in response to HS during this period and assess the effect of oral progestin (altrenogest; **ALT**) supplementation. Postpubertal gilts (*n* = 42) were artificially inseminated during behavioral estrus (*n* = 28) or were kept cyclic (*n* = 14), and randomly assigned to thermal neutral (**TN**; 21 ± 1 °C) or diurnal HS (35 ± 1 °C for 12 h/31.6 ± 1 °C for 12 h) conditions from day 3 to 12 postestrus (**dpe**). Seven of the inseminated gilts from each thermal treatment group received ALT (15 mg/d) during this period. Using quantitative PCR, transcript abundance of HSP family A (Hsp70) member 1A (*HSPA1A*, *P* = 0.001) and member 6 (*HSPA6*, *P* < 0.001), and HSP family B (small) member 8 (*HSB8*, *P* = 0.001) were increased while HSP family D (Hsp60) member 1 (*HSPD1*, *P* = 0.01) was decreased in the endometrium of pregnant gilts compared to the cyclic gilts. Protein abundance of HSPA1A decreased (*P* = 0.03) in pregnant gilt endometrium due to HS, while HSP family B (small) member 1 (HSPB1) increased (*P* = 0.01) due to HS. Oral ALT supplementation during HS reduced the transcript abundance of HSP90α family class B member 1 (*HSP90AB1*, *P* = 0.04); but HS increased *HSP90AB1* (*P* = 0.001), HSPA1*A* (*P* = 0.02), and *HSPA6* (*P* = 0.04) transcript abundance irrespective of ALT. ALT supplementation decreased HSP90α family class A member 1 (HSP90AA1, *P* = 0.001) protein abundance, irrespective of thermal environment, whereas ALT only decreased HSPA6 (*P* = 0.02) protein abundance in TN gilts. These results indicate a notable shift of HSP in the porcine endometrium during the peri-implantation period in response to pregnancy status and heat stress.

## Teaser

Heat stress (HS) causes economic losses to the swine industry by compromising reproduction during summer months. This research study reports the changes observed in porcine endometrial heat shock protein (HSP) milieu during the peri-implantation period in response to pregnancy status and in conditions of HS.

## Introduction

Environmental induced hyperthermia during the warm summer months compromises efficient commercial swine production (St-Pierre et al., 2003; Auvigne et al., 2010), in large part due to its multifaceted impact on reproduction (Baumgard et al., 2012; Ross et al., 2017; Mayorga et al., 2020). The highly malleable endometrium undergoes diverse adaptations during the peri-implantation period such as tissue remodeling, cellular apoptosis, and angiogenesis in response to physiological cues regulated by ovarian steroids, cytokines, and growth factors, to ultimately facilitate conceptus implantation (Bazer et al., 2010; Chae et al., 2011; Franczak et al., 2013). Disturbances in maternal homeostasis, caused by stressors such as extreme heat or nutritional imbalances, can perturb the conceptus–endometrial crosstalk and result in defective development precluding pregnancy establishment (Omtvedt et al., 1971; Tast et al., 2002; Hansen, 2009; Kraeling and Webel, 2015).

The overarching influence of heat stress (**HS**) on infecundity (De Rensis et al., 2017; Ross et al., 2017) includes disturbed follicular growth (Badinga et al., 1993; Bertoldo et al., 2010), altered ovarian phosphatidylinositol-3 kinase and steroidogenic signaling (Nteeba et al., 2015), reduced corpora lutea size (Bidne et al., 2019), autophagic changes in follicular development (Hale et al., 2017), dysfunctional steroidogenesis (Bertoldo et al., 2011), altered endometrial cell function (Gross et al., 1989), and as an effect of endotoxemia (Bidne et al., 2018a). Thus, identifying mitigation strategies to improve fertility during HS would improve farm economics and assist with efforts to increase the sustainability of the pork industry (Cottrell et al., 2015; Cabezón et al., 2017; De Rensis et al., 2017; Biggs et al., 2020). Altrenogest (**ALT**), a progesterone analog developed for gilt estrus synchronization (Kraeling and Webel, 2015), has been assessed for its ability to improve ovulation and conception rate, total number of piglets born alive, and litter size (Martinat-Botte et al., 1995; van Leeuwen et al., 2011; Wang et al., 2018). Progesterone administered during early gestation improves embryo growth in cattle (Garrett et al., 1988; Carter et al., 2008), sheep (Satterfield et al., 2006), and pigs (Muro et al., 2020). ALT supplementation also advances conceptus development when provided during the peri-implantation period (Romoser et al., 2022).

As molecular chaperones, heat shock proteins (**HSP**) are involved in transport, correct folding and assembly of polypeptides, as well as disassembly and degradation of proteins; tasks essential for cellular homeostasis (Witkin and Linhares, 2010; Evgen’Ev et al., 2014a). The gene sequences of HSP are highly conserved across organisms from bacteria to plants to mammals, as they are essential for cell survival (Sørensen et al., 2003) and their function is more critical under conditions of stress (Burel et al., 1992). HSP are categorized into families based on their molecular weight and vary considerably in their functions, cellular abundance, and subcellular localization (Richter et al., 2010; Evgen’Ev et al., 2014b). In addition to the aforementioned functions, HSP are also involved in cell proliferation and differentiation, as well as in regulating apoptosis and actin filament organization; processes critical to endometrial remodeling and conceptus development in early pregnancy (Welch et al., 1993; Neuer et al., 2000; Seibert et al., 2019; Jee et al., 2021).

Even in the absence of stress, mammalian cells constitutively express certain HSP, known as cognate HSP, which are involved in protein folding, transfer of proteins across membranes, and developmental processes such as embryogenesis (Burel et al., 1992). During normal gestation endometrial HSP abundance is variable, observed as increased or decreased abundance depending on the stage of gestation and HSP class (Tabibzadeh et al., 1996; Chae et al., 2011; Jee et al., 2021). Inducible HSP are produced in response to threats such as thermal or oxidative stress, ischemia, and bacterial or viral infections (Burel et al., 1992; Neuer, 2000). Endotoxemia due to bacterial lipopolysaccharide (**LPS**) presence negatively affects female fertility (Bidne et al., 2018a) similar to that observed as an effect of HS (Mayorga et al., 2020), potentially because HSP are involved in cellular pathways responsive to both LPS and HS.

Endometrial HSP abundance in pigs in response to HS during the peri-implantation period is not well characterized. Hence, study goals were to evaluate the transcript and protein abundance of endometrial HSP during the peri-implantation period in postpubertal gilts and assess whether they were altered by oral ALT supplementation. The hypothesis was that environmental HS would alter the endometrial HSP milieu during the peri-implantation period in pigs.

## Materials and Methods

### Ethics statement

All animal procedures conducted in this study were approved by the Iowa State University Institutional Animal Care and Use Committee.

### Experimental design

**Figure 1.**
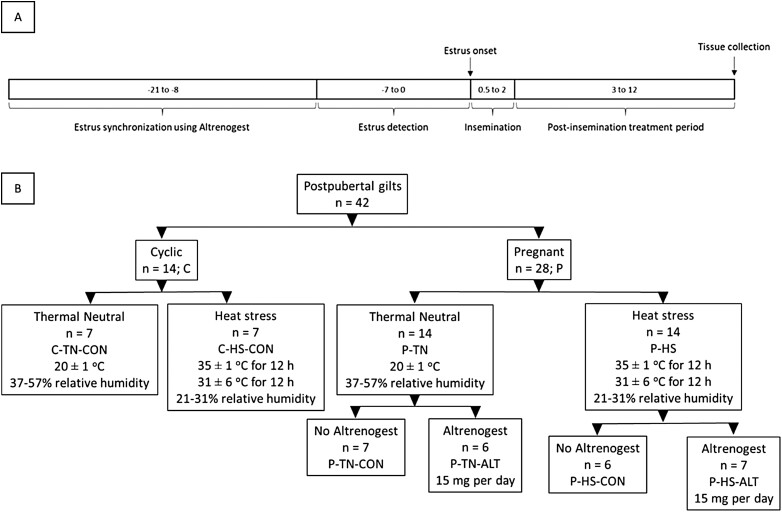
Experimental design for the study. (A) Postpubertal gilts (*n* = 42) were estrus synchronized and checked for signs of estrus. (B) During behavioral estrus, these gilts were either artificially inseminated (*n* = 28; P) or were not inseminated (*n* = 14; C), and then randomly assigned to thermal neutral (*n* = 7 [C-TN] and *n* = 14 [P-TN]) or diurnal heat stress (*n* = 7 [C-HS] and *n* = 14 [P-HS]) conditions from day 3 to 12 postestrus. The inseminated animals from each thermal treatment group were either maintained without ALT supplementation (*n* = 7 [P-TN-CON] and *n* = 7 [P-HS-CON]) or supplemented with ALT (15 mg/d; *n* = 7 [P-TN-ALT] and *n* = 7 [P-HS-ALT]), from days 3 to 12 postestrus. All the cyclic animals were maintained without ALT supplementation (C-TN-CON and C-HS-CON). One gilt each from the P-TN-ALT and the P-HS-CON groups was excluded from the current study, as they were observed to be nonpregnant, confirmed by the absence of conceptus in the uterus.

Forty-two postpubertal gilts were estrus synchronized using oral ALT supplementation (15 mg/d; Matrix; Merck Animal Health, NJ) and checked for signs of estrus, as depicted in the experimental design (Figure 1A) and described previously (Romoser et al., 2022). During behavioral estrus, 28 gilts were artificially inseminated (pregnant, P) and 14 were not inseminated (cyclic, C). They were then randomly assigned (Figure 1B) to thermal neutral (21 ± 1 °C; *n* = 7 [C-TN] and *n* = 14 [P-TN]) or diurnal HS (35 ± 1 °C for 12 h/31.6 ± 1 °C for 12 h; *n* = 7 [C-HS] and *n* = 14 [P-HS]) conditions from days 3 to 12 postestrus (**dpe**). In each thermal treatment group, half of the inseminated animals received ALT (*n* = 7 [P-TN-ALT] and *n* = 7 [P-HS-ALT]), whereas the others were maintained without ALT (*n* = 7 [P-TN-CON] and *n* = 7 [P-HS-CON]), from 3 to 12 dpe. All cyclic animals were maintained without ALT supplementation (C-TN-CON and C-HS-CON).

Pregnancy was confirmed by the presence of conceptuses within the uterus during sample collection. One gilt from the P-TN-ALT group and one from the P-HS-CON group were excluded from the current study, as they were observed to be nonpregnant based on conceptus absence in the uterus.

A measured response to thermal treatments was assessed using defined parameters such as change in rectal temperature, respiration rate, and feed intake, as previously described (Romoser et al., 2022). Baseline measurements collected during the initial synchronization period allowed us to confirm a definitive effect of HS, observed as an increase in rectal temperature and respiration rate, and consequent decrease in feed intake in the pigs under HS when compared with those maintained in TN conditions (Romoser et al., 2022).

### Sample collection

Individual uterine horns were separately flushed with sterile phosphate-buffered saline as described previously (Ross et al., 2003a; Romoser et al., 2022). Conceptuses were separated into cryovials and snap frozen, and uterine flush fluid was collected in sterile 15-mL conical tubes kept on ice, for a separate study. Individual uterine horns were dissected lengthwise along the antimesometrial border, endometrial tissue was separated from the myometrium, flash frozen in liquid nitrogen, and then stored at −80 °C, until further processing.

### RNA isolation and quantitative one-step RT-PCR

Total RNA was isolated from frozen endometrial tissue using a standard phenol:chloroform (TRIzol, Invitrogen, Waltham, MA) extraction protocol followed by ethanol precipitation, according to the manufacturer’s instructions. All samples were treated with deoxyribonuclease I (Ambion, Austin, TX) to eliminate any genomic DNA amplification during downstream processes. Concentration and quality of RNA were determined by a NanoDrop spectrophotometer (ThermoFisher Scientific, Waltham, MA) and agarose gel electrophoresis before being utilized for quantitative real-time reverse transcription polymerase chain reaction (qPCR).

Relative transcript abundance was quantified using 25 ng of total RNA using the QuantiTect SYBR Green RT-PCR Kit (Qiagen, Hilden, Germany) on an Eppendorf MasterCycler (Eppendorf, Hamburg, Germany). PCR primer sequences (Table 1) for each target gene were used in thermal cycling conditions for SYBR Green detection as follows: 50 °C for 30 min, 95 °C for 15 min, followed by 40 cycles at 94 °C for 15 s, annealing at 57 to 60 °C for 30 s, and a final step at 72 °C for 30 s followed by fluorescent data acquisition. Product specificity was confirmed with melting curve analyses subsequent to completion of the thermal cycling protocol. The amplification efficiency was between 90% and 105% for all primer sets. The reference gene chosen as an endogenous normalization control, glyceraldehyde-3-phosphate dehydrogenase (***GAPDH***), was not affected by either treatment. Relative quantification of transcript abundance was calculated using the comparative cycle threshold (***C***_***t***_) method. Briefly, the Δ*C*_*t*_ value was determined by subtracting the *GAPDH* C_t_ value from its respective target *C*_*t*_ for each sample. ΔΔ*C*_*t*_ was calculated by subtracting Δ*C*_*t*_ values for each sample from the single greatest sample Δ*C*_*t*_; hence using the sample with the lowest expression as an arbitrary constant (Ross et al., 2009). Relative fold change for each sample was calculated using the equation 2^ΔΔCt^.

**Table 1.** Primer sequences used to assess transcript abundance in porcine endometrial tissue

Gene	Gene name	Primer sequence, 5´ → 3´
*GAPDH*	Glyceraldehyde 3-phosphate dehydrogenase	F: TGGTGAAGGTCGGAGTGAAC
		R: GAAGGGGTCATTGATGGCGA
*HSP90AA1*	Heat shock protein 90 α family class A member 1	F: CTGGTCAAGAAATGCTTGGAG
		R: TGGTCCTTGGTCTCACCTGT
*HSP90AB1*	Heat shock protein 90 α family class B member 1	F: AACACTGCGGTCAGGGTATC
		R: ACATTCCCTCTCCACACAGG
*HSPA1A*	Heat shock protein family A (Hsp70) member 1A	F: AGGCGGACAAGAAGAAGGTG
		R: CCGCTGATGATGGGGTTACA
*HSPA6*	Heat shock protein family A (Hsp70) member 6	F: GAATCCGCAGAATACCGTGT
		R: TCCGCAGTCTCCTTCATCTT
*HSPD1*	Heat shock protein family D (Hsp60) member 1	F: ATGCTTCGATTACCCGCAGT
		R: AAGCCCGAGTGAGATGAGGA
*HSPB1*	Heat shock protein family B (small) member 1	F: TCGAAAATACACGCTGCCCC
		R: TTCCGGGCTTTTCCGACTTT
*HSPB8*	Heat shock protein family B (small) member 8	F: GTCTGGCAAACACGAGGAGA
		R: TGGGGAAAGCGAGGCAAATA

### Western blot analysis

Frozen endometrial tissue was homogenized in radioimmunoprecipitation assay lysis buffer (50 mM Tris–HCl at pH 8.0, 150 mM NaCl, 0.1% sodium dodecyl sulfate (**SDS**), 1 % Nonidet P-40, 0.5% sodium deoxycholate) with protease inhibitor cocktail, incubated on ice for 30 min, and centrifuged at 10,000 revolutions per minute at 4 °C for 15 min. The supernatant was separated and utilized for total protein quantification using Pierce BCA Protein Assay Kit (ThermoFisher Scientific, Waltham, MA) on a microplate spectrophotometer (BioTek, Agilent, Santa Clara, CA). Samples (4 µg/µL) were denatured in loading buffer (50 mM Tris–HCl pH 6.8, 2% SDS, 10% glycerol, 1% beta-mercaptoethanol, 12.5 mM ethylenediaminetetraacetic acid, 0.02% bromophenol blue) at 95 °C for 5 min, and then stored at −80 °C. Protein samples (40 µg) were loaded into a 4 to 20% Criterion TGX Stain-Free Protein Gel (Bio-Rad, Hercules, CA), separated at 100 volts for 90 min at room temperature (**RT**), and transferred to a nitrocellulose membrane using the iBlot 2 dry blotting system (ThermoFisher Scientific, Waltham, MA) at 25 V for 6 min at RT. Consistent protein loading was confirmed by Ponceau S staining of the nitrocellulose membranes. Subsequently, membranes were washed three times in Tris-buffered saline with 0.1% Tween20 (**TBST**) for 5 min each at RT, and then incubated in blocking solution (5% milk in TBST) for 1 h at RT with gentle shaking. Membranes were then incubated at 4 °C overnight with primary antibodies specific for HSP90AA1 (1:1,000; 8165; Cell Signaling Technology, Danvers, MA), HSP90AB1 (1:1,000; 7411; Cell Signaling Technology, Danvers, MA), HSPA6 (1:1,000; NBP1-85949; Novus Biologicals, Centennial, CO), HSPA1A (1:1,000; NB110-96427; Novus Biologicals, Centennial, CO), HSPB1 (1:1,000; NBP2-25149; Novus Biologicals, Centennial, CO), and HSPD1 (1:1,000; NBP2-32973; Novus Biologicals, Centennial, CO) diluted in 5% milk in TBST. Negative controls such as normal rabbit IgG (1:1,000; 2729; Cell Signaling Technology, Danvers, MA), normal mouse IgG (1:1,000; 5415; Cell Signaling Technology, Danvers, MA), or excluding the primary antibody incubation were tested on a pool of all individual samples, to confirm specificity of primary antibodies for HSP assessed. Membranes were washed in TBST three times for 10 min at RT with gentle shaking, and then incubated in the appropriate horseradish peroxidase (**HRP**)-tagged secondary antibody (1:10,000) for 1 h at RT. After three more washes in TBST for 10 min at RT, membranes were incubated in HRP substrate (MilliporeSigma, St. Louis, MO) for 5 min in the dark. Excess substrate was decanted, and images were captured using an Azure c600 Imager (Azure Biosystems, Dublin, CA). Densitometric analysis was conducted using Image Studio Lite (LI-COR Biosciences, Lincoln, NE) to quantify band intensities for proteins of interest and normalized to Ponceau S band intensities for each blot.

### Statistical Analysis

The six treatment groups were separated to conduct two major comparisons of transcript and protein abundance. The first comparison evaluated the main effects of the two independent variables, pregnancy status and thermal treatment, as well as their interaction, on the dependent variable (HSP abundance). This analysis included the cyclic (**C**) and pregnant (**P**) gilts from both the TN and HS thermal treatment groups, and none of these gilts were supplemented with ALT postinsemination. This comparison is a 2 (pregnancy status: C or P) by 2 (thermal treatment: TN or HS) factorial design.

The second comparison evaluated the main effects of the two independent variables, ALT supplementation and thermal treatment, as well as their interaction, on the dependent variable (HSP abundance). This analysis included only the pregnant (P) gilts, exposed to TN or HS thermal conditions, that were treated with (ALT) or without ALT supplementation (CON) postinsemination. This comparison is a 2 (altrenogest supplementation: CON or ALT) by 2 (thermal treatment: TN or HS) factorial design.

Statistical analyses were conducted using GraphPad Prism 9.0.0 (GraphPad Software, San Diego, CA) and results are presented as the LS means ± SEM. After a normality check, the numerical data for transcript and protein abundance were assessed using a two-way ANOVA test for each treatment group comparison as described above. The main effects of the factors and their interaction, for each treatment group comparison, were assessed. This was followed by a post hoc Tukey’s analysis to discern differences between individual treatment groups. Biological significance was considered if *P* ≤ 0.05 and a tendency for biological meaning was considered when 0.05 < *P* ≤ 0.10.

## Results

### Pregnancy status has a greater influence on the endometrial transcript abundance of specific HSP than thermal treatment

**Figure 2.**
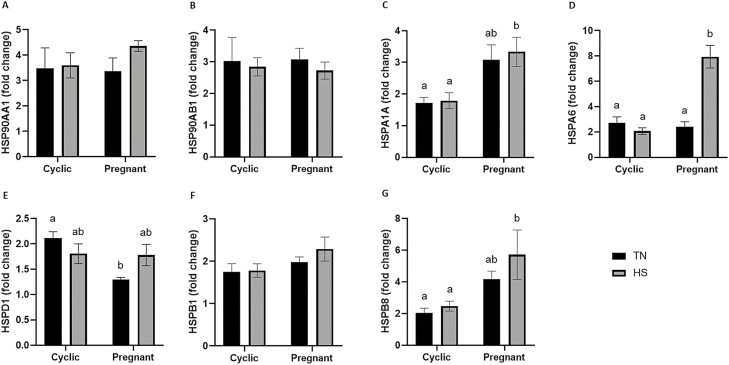
Effect of pregnancy and/or thermal treatment on endometrial transcript abundance of heat shock proteins. Endometrium from cyclic and pregnant gilts maintained under thermal neutral or heat stress conditions were assessed for changes in specific HSP transcript abundance using qPCR. No effect of pregnancy status or thermal treatment on (A) *HSP90AA1* and (B) *HSP90AB1* was observed. (C) *HSPA1A* was elevated in the pregnant endometrium irrespective of thermal treatment. (D) *HSPA6* was increased in the pregnant endometrium under HS when compared with the other treatment groups. (E) Pregnancy status reduced *HSPD1* transcript abundance whereas HS had no effect. Neither pregnancy status nor thermal treatment influenced (F) *HSPB1* transcript abundance, but (G) *HSPB8* was elevated in the pregnant endometrium under HS conditions. Normalized transcript abundances are reported as fold change. Different lowercase letters indicate statistical significance between treatment groups; *P* ≤ 0.05.

In the first set of comparisons (Figure 2A–G), cyclic and pregnant gilts were included (neither group supplemented with ALT postinsemination) while in TN or HS environments during the peri-implantation period. The endometrial transcript abundance of specific HSP was assessed as a main effect of pregnancy status or HS, or due to an interaction of both factors. Pregnant gilts had elevated *HSPA1A* transcript abundance when compared with the cyclic counterparts, whether maintained in TN (1.8-fold; *P* = 0.05) or HS (1.9-fold; *P* = 0.03; Figure 2C) conditions. Moreover, pregnant gilts had an elevated *HSPA1A* transcript abundance during HS (1.9-fold; *P* = 0.02) when compared with the cyclic TN counterparts. Both pregnancy status (*P* < 0.001) and HS (*P* < 0.001), individually and as an interaction (*P* < 0.001), increased *HSPA6* transcript abundance (Figure 2D). The pregnant endometrium had elevated (3.8-fold; *P* < 0.001) *HSPA6* transcript abundance when compared with the cyclic endometrium, when both were maintained in HS. Similarly, HS increased *HSPA6* transcript abundance in the pregnant endometrial, compared to the pregnant (3.3-fold; *P* < 0.001) as well as the cyclic (2.9-fold; *P* < 0.001) endometrium, maintained in TN conditions. Endometrial *HSPD1* transcript abundance (Figure 2E) was decreased in pregnant gilts maintained in TN conditions when compared with their cyclic counterparts (1.6-fold; *P* = 0.004) and there was a tendency for a reduction in *HSPD1* compared to cyclic HS gilts (1.4-fold; *P* = 0.1). Pregnancy status increased (*P* = 0.001) *HSPB8* transcript abundance (Figure 2G), with pregnant gilts having increased abundance when compared with cyclic gilts (2.3-fold; *P* = 0.02) when both were maintained in HS conditions. Pregnancy status and HS in combination, elevated *HSPB8* transcript abundance compared to the TN cyclic group (2.8-fold; *P* = 0.01). Transcript abundance of *HSP90AA1* (Figure 2A), *HSP90AB1* (Figure 2B) and *HSPB1* (Figure 2F) were not affected by pregnancy status nor HS.

### Heat stress increased endometrial transcript abundance of specific HSP regardless of altrenogest supplementation

**Figure 3.**
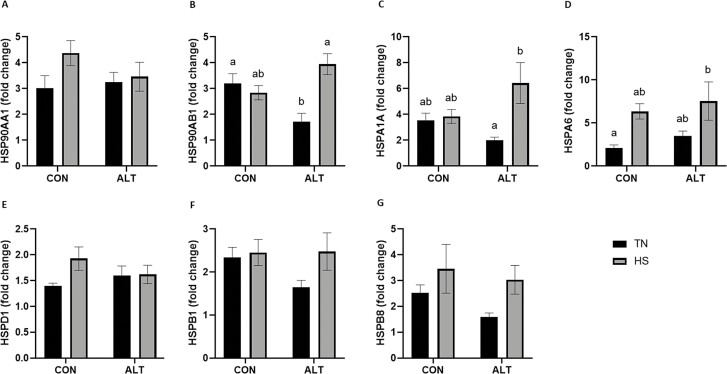
Effect of altrenogest and/or thermal treatment on endometrial transcript abundance of heat shock proteins. Endometrium from pregnant gilts either supplemented with altrenogest or not (postinsemination), and maintained under thermal neutral or heat stress conditions during the peri-implantation period were assessed for changes in specific HSP transcript abundance using qPCR. Neither ALT supplementation nor thermal treatment demonstrated an effect on (A) *HSP90AA1* transcript abundance. (B) *HSP90AB1* abundance was reduced as a result of ALT supplementation under TN conditions; an effect reversed by HS in spite of ALT supplementation. HS also increased (C) *HSPA1A* and (D) *HSPA6* transcript abundance, in spite of ALT supplementation. No effects of either treatment were observed on (E) *HSPD1*, (F) *HSPB1*, and (G) *HSPB8* transcript abundances. Normalized transcript abundances are reported as fold change. Different lowercase letters indicate statistical significance between treatment groups; *P* ≤ 0.05.

The second set of comparisons (Figure 3A–G) included only pregnant gilts either supplemented with ALT postinsemination or not, while in TN or HS conditions during the peri-implantation period. Endometrial transcript abundance of specific HSP was evaluated as a main effect of HS or ALT supplementation, or as an interaction of both factors. ALT supplemented gilts had decreased *HSP90AB1* transcript abundance (1.9-fold; *P* = 0.04; Figure 3B) compared to their nonsupplemented counterparts when both groups were maintained in TN conditions. HS increased *HSP90AB1* (2.3-fold; *P* = 0.001; Figure 3B) abundance regardless of ALT supplementation. Similarly, increased *HSPA1A* (3.2-fold; *P* = 0.02; Figure 3C) transcript abundance was observed in HS compared to TN gilts, when both groups were supplemented with ALT. Though HS increased (*P* = 0.01) *HSPA6* transcript abundance overall (Figure 3D), this increase (3.6-fold; *P* = 0.04) was due to the combined effect of ALT supplementation and HS compared to the nonsupplemented TN gilts. The transcript abundance of *HSP90AA1* (Figure 3A), *HSPD1* (Figure 3E), *HSPB1* (Figure 3F), and *HSPB8* (Figure 3G) were not affected by HS or ALT.

### Endometrial HSP protein abundance is selectively influenced by pregnancy status and thermal treatment

**Figure 4.**
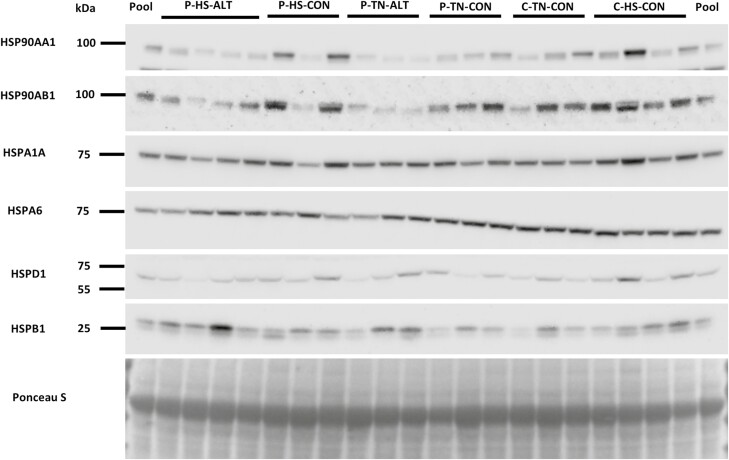
Assessment of heat shock protein abundance in the endometrial samples by Western blotting. Representative figure of immunoblotting of endometrial protein lysates for each treatment group using antibodies directed toward HSP90AA1, HSP90AB1, HSPA1A, HSPA6, HSPD1, and HSPB1. Ponceau S stained blots were imaged to ascertain uniform loading and bands thus obtained were utilized for loading control normalization of specific target proteins. The samples from different treatment groups were uniformly distributed across two gels and pooled samples were used on both gels to allow normalization and unbiased comparisons of band densities across both gels.

**Figure 5.**
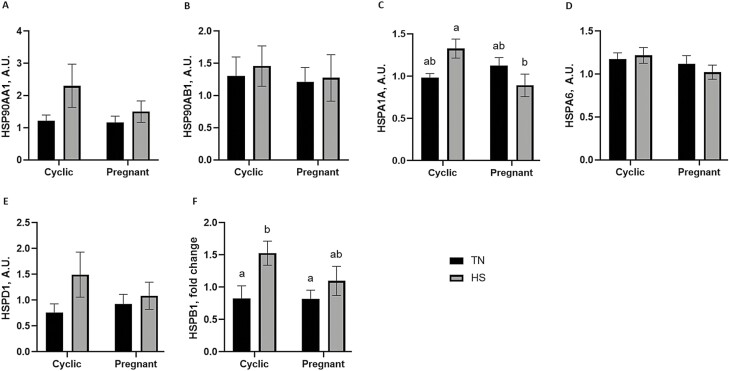
Effect of pregnancy and/or thermal treatment on endometrial protein abundance of heat shock proteins. Endometrium from cyclic and pregnant gilts maintained under thermal neutral or heat stress conditions were assessed for changes in HSP protein abundance using densitometric analysis of immunoblots. Neither pregnancy status nor thermal treatment affected (A) HSP90AA1 or (B) HSP90AB1 abundance. (C) HSPA1A protein abundance was decreased in the pregnant endometrium (in spite of HS), but no effect was observed on (D) HSPA6 protein abundance. Pregnancy status and thermal treatment had no effect on (E) HSPD1 abundance, but HS increased (F) HSPB1 protein abundance in the cyclic gilts. Different lowercase letters indicate statistical significance between treatment groups; *P* ≤ 0.05.

Western blotting analyses assessed the protein abundance of specific HSP in the endometrium (Figure 4). The main effect of pregnancy status or HS, or an effect due to an interaction of both factors on these HSP (Figure 5A–F) were evaluated by comparing the cyclic and pregnant gilts (neither group supplemented with ALT) while in TN or HS environments during the peri-implantation period. HS tended to increase (1.4-fold; *P* = 0.09) HSPA1A protein abundance in the cyclic gilts, although HSPA1A protein was decreased (1.5-fold; *P* = 0.03; Figure 5C) in pregnant endometrium (when compared with cyclic). HS increased (*P* = 0.01; Figure 5F) the endometrial HSPB1 protein abundance, which was elevated in the cyclic animals in HS when compared with their TN counterparts (1.9-fold; *P* = 0.05) as well as the TN pregnant gilts (1.9-fold; *P* = 0.05). Neither pregnancy status nor HS affected the protein abundance of HSP90AA1 (Figure 5A), HSP90AB1 (Figure 5B), HSPA6 (Figure 5D), and HSPD1 (Figure 5E).

### Altrenogest supplementation decreased endometrial protein abundance of specific HSP irrespective of thermal treatment

**Figure 6.**
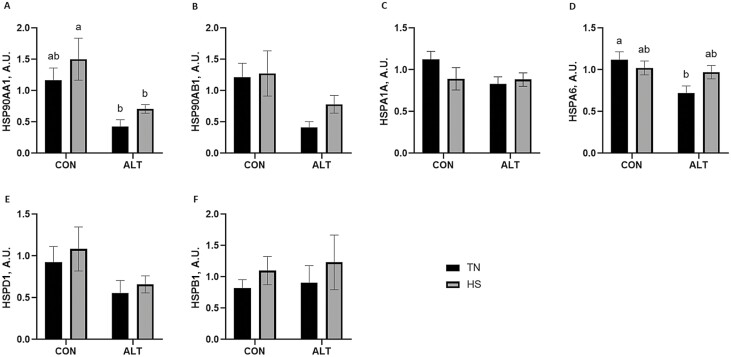
Effect of altrenogest and/or thermal treatment on endometrial protein abundance of heat shock proteins. Endometrium from pregnant gilts either supplemented with ALT (postinsemination) or not and maintained under thermal neutral or heat stress conditions during the peri-implantation period were assessed for changes in specific HSP protein abundance using densitomentric analysis of immunoblots. ALT supplementation decreased (A) HSP90AA1 protein abundance irrespective of thermal treatment, with a tendency to decrease (B) HSP90AB1 abundance. Neither ALT supplementation nor HS influenced (C) HSPA1A abundance, but ALT reduced (D) HSPA6 protein abundance in the gilts maintained under TN conditions. (E) HSPD1 and (F) HSPB1 protein abundance were unchanged by either treatment. Different lowercase letters indicate statistical significance between treatment groups; *P* ≤ 0.05.

The main effect of ALT supplementation or HS, or an effect due to an interaction of both factors, on protein abundance of specific HSP (Figure 6A–F) was evaluated in the pregnant gilts either supplemented with ALT postinsemination or not, while in TN or HS environments during the peri-implantation period. Overall, ALT supplementation decreased (*P* = 0.001) HSP90AA1 protein abundance (Figure 6A) with a 2.7-fold decrease (*P* = 0.07) in TN gilts and a 2.1-fold decrease in HS gilts (*P* = 0.04). Moreover, HSP90AA1 protein abundance was decreased in the ALT supplemented TN gilts (3.5-fold; *P* = 0.01) compared to the non-ALT supplemented HS gilts (Figure 6A). Overall, ALT supplementation decreased (*P* = 0.01) HSP90AB1 protein abundance, and there was a tendency for HSP90AB1 to be decreased in TN gilts compared to nonsupplemented TN (2.9-fold; *P* = 0.08) and nonsupplemented HS (3.1-fold; *P* = 0.07) gilts. Similarly, HSPA6 abundance was lower (1.6-fold; *P* = 0.02) in TN gilts supplemented with ALT when compared with the nonsupplemented TN gilts (Figure 6D). Neither HS nor ALT supplementation impacted the protein abundance of HSPA1A (Figure 6C), HSPD1 (Figure 6E), or HSPB1 (Figure 6F) in the pregnant animals.

## Discussion

Peri-implantation events such as apposition and adhesion between the maternal endometrial surface and the elongating porcine conceptus are critical prerequisites for fetal and placental development (Bazer et al., 2010; Bauersachs and Wolf, 2012; Geisert et al., 2015). Normally, pregnancy establishment requires homeorhetic shifts in many tissues to support the demands of placental and fetal development. Environmental stress, such as HS, may compromise the flexibility of specific tissues to effectively accommodate pregnancy establishment and support of fetal growth.

Susceptibility of the early developing embryo to thermal stress has been reported (Tompkins et al., 1967; Ealy et al., 1993; van Wettere et al., 2021). Mammalian embryos in the very early stages of development are characterized by a lack of induced HSP synthesis, an inability reflective of the absence of embryonic gene transcription, and hence depend on endometrial support for an HSR. HSP presence has been reported in the endometrium of women (Tabibzadeh et al., 1996; Shah et al., 1998; Jee et al., 2021), mares (Camacho Benítez et al., 2021), dairy cattle (Bai et al., 2020), and pigs (Chae et al., 2011). The abundance of HSP, both cognate and inducible forms, can be altered during physiological processes (cellular growth or differentiation), in pathological conditions (infections or cancerous) or as a result of environmental stressors, such as thermal or nutritional stress (Burel et al., 1992; Neuer et al., 2000; Seibert et al., 2019).

This study evaluated the abundance of HSP90 (HSP90AA1 and HSP90AB1), HSP70 (HSPA1A and HSPA6), HSP60 (HSPD1), and small HSP (HSPB) in the porcine endometrium during the peri-implantation period. We demonstrate how a physiological event such as pregnancy and an environmental stressor such as HS, individually and in combination, alter the endometrial abundance of HSP. Moreover, supplementation of pregnant gilts subjected to HS with ALT also altered the abundance of these HSP in the porcine endometrium.

Dynamic changes in endometrial HSP abundances have been reported during the peri-implantation period, indicative of their involvement in cellular events essential for implantation (Tabibzadeh et al., 1996; Neuer, 2000; Yuan et al., 2009). HSP have an important role in the controlled inflammatory response required for conceptus implantation and trophoblast growth (Jee et al., 2021). In the current study, the pregnant gilts had higher endometrial *HSPA1A* abundance when compared with their cyclic counterparts when both groups were exposed to HS. Interestingly, the HSPA1A protein abundance was distinctively lower in the pregnant group when compared with the cyclic group when both groups experienced HS. A similar trend was observed with an increased *HSPA6* transcript abundance in pregnant pigs maintained under HS conditions compared to the other groups, while the protein abundance stayed the same between treatment groups. These observations indicate a faster turnover and/or release of this cytoprotective protein into the uterine microenvironment. Interestingly, Romoser et al., (2022) have recently reported that HS during the peri-implantation period did not affect conceptus elongation rate. This could be ascribed to a potential protective effect due to the interaction of conceptus tissue with the endometrial HSP released into the intrauterine microenvironment.

Release of HSP into extracellular compartments is involved with activation of immune cells, intercellular communication, and induction of signaling pathways (De Maio, 2011). Moreover, HSP70 causes an inflammatory effect by inducing production of cytokines such as tumor necrosis factor alpha, interleukin 1-beta and interleukin 6 in immune cells (Asea and De Maio, 2007). Immune modulation during the peri-implantation period mediates communication between the developing conceptus and endometrium (Ross et al., 2003b; Mathew et al., 2016). Modulation of the uterine immune microenvironment also serves as a priming event during stress (De Maio and Vazquez, 2013). Thus, the HSP released into the uterine microenvironment could potentially be a cytoprotective chaperokine during HS, by exerting immunoregulatory effects mediated via Toll-like receptor-2 (TLR2) and TLR4 (Asea, 2003).

Multiple HSP regulate cytoprotective events within different apoptotic pathways to either aid cell survival or cell recovery following damaging stimuli. Typically HSPD1 is present in the mitochondria and is released during stress to optimize caspase activation (Beere, 2004). In the current study, the endometrial transcript abundance of *HSPD1* was lower in the pregnant TN group compared to their cyclic counterparts, but the protein abundance was not different. The active endometrial remodeling occurring during this period in the pregnant uterus might explain these differences. Similarly, small intracellular HSP which are involved in maintenance of cell survival as well as stabilization of cytoskeletal components (Carra et al., 2017), are elevated in reproductive tissues under the influence of steroid hormones (de Thonel et al., 2012; Jee et al., 2021), a scenario that could explain the elevated *HSPB8* transcript abundance observed in the pregnant gilts compared with their cyclic counterparts.

Heat stress induces gene expression involved in apoptosis (Beere, 2004; Jin et al., 2007) and autophagy (Hale et al., 2017). In the current study, HS elevated *HSPD1* transcript abundance in the pregnant endometrium. A similar HS-induced HSPD1 increase was reported in the porcine ovary (Seibert et al., 2019), indicating a stress response within the mitochondria, often correlated with apoptosis. Different apoptosis-related factors are involved in the tight regulation of endometrial apoptosis correlated with the hormonally dependent changes in endometrial cell transformation (Antsiferova, 2016) during the peri-implantation period, which might explain the differences observed in the current study.

Heat stress negatively impacts reproduction via multiple mechanisms, which ultimately manifests as seasonal infertility (De Rensis et al., 2017; Ross et al., 2017). The HS-induced effect on corpora lutea size (Bidne et al., 2019) could be involved in early pregnancy loss due to luteal insufficiency. The pig depends on a functional corpus luteum as the progesterone source, which plays a critical role in maintaining pregnancy in other species as well (Geisert et al., 2015; Lonergan et al., 2016; Spencer et al., 2016). Progesterone is required during endometrial transformation around the time of conceptus implantation, as it suppresses proliferation and induces cell differentiation. Low progesterone levels are associated with underdeveloped conceptuses (Spencer et al., 2016) and exogenous supplementation of progestogens has been shown to positively influence reproductive parameters (Martinat-Botte et al., 1995; van Leeuwen et al., 2011; Muro et al., 2021). Moreover, Romoser et al. (2022) have recently reported the potentially beneficial effects of ALT supplementation on conceptus development in HS gilts.

Progesterone is reported to interact with and regulate the synthesis of uterine tissue HSP to modulate responsiveness of the endometrium to steroid hormones (Bagchi et al., 1991; Padwick et al., 1994; Neuer et al., 2000). Although HSP90AA1, a thermal stress-inducible HSP, was not affected by either HS nor pregnancy status, ALT decreased HSP90AA1 and HSP90AB1 protein abundance. The interesting observation here was that during HS, ALT supplemented gilts had half the HSP90 protein abundance compared to the nonsupplemented group. HSP90 is a co-factor for steroid hormone receptors and is released from the receptor complex upon ligand (hormone)-receptor interaction (Bagchi et al., 1991; Dhamad et al., 2016) leading to downstream hormone activity. This could help explain why ALT caused a reduction in HSP abundance, if it were being released into the extracellular space as reported earlier (De Maio, 2011).

The differential response of HSP abundance with respect to pregnancy status or HS observed in this study could be related to a cell specific response, which was difficult to discern in the endometrial samples collected in this study as the epithelial and stromal cells of the endometrium were not separately evaluated. A similar cell specific response to HS has been demonstrated in the porcine ovary (Pennarossa et al., 2012). It is proposed that HSP provide beneficial cytoprotective effects when induced prior to an inflammatory insult, but thereafter can have deleterious effects as inflammation progresses (Yu et al., 2007). Gilts in this study were exposed to HS from 2 to 12 dpe and could potentially have had a different HSP milieu if assessed earlier in the study timeline. It also remains unclear whether these differences in HSP abundance are a direct effect of HS on the endometrial tissue or as an indirect effect of endotoxins released into the circulation, which in turn induce endometrial HSP. Endotoxemia due to circulating LPS affects female reproduction in many ways (Bidne et al., 2018a) causing impaired estradiol synthesis in granulosa cells (Li et al., 2017), induction of pro-inflammatory ovarian environment (Dickson et al., 2016), embryonic DNA damage and pregnancy failure in mice (Jaiswal et al., 2013), pregnancy loss due to progesterone deficiency in mares (Daels et al., 1991), and impaired follicular growth and steroidogenesis in cattle (Roth and Wolfenson, 2016). Both HS (Dickson et al., 2018) and LPS (Bidne et al., 2018b) alter ovarian signaling during follicular development in postpubertal gilts. Moreover, HSP abundance in the porcine ovary in response to HS and LPS is similar depending on the stage of estrous cycle (Seibert et al., 2019) warranting further examination of the endometrial HSP response observed in the current study.

## Conclusion

Constitutive endometrial HSP expression in pregnant gilts and induced expression of HSP due to thermal stress may both represent an essential requirement for successful conceptus growth in an adverse environment. Overexpression of HSP, identified with ALT supplementation, is probably to the benefit of the developing conceptus too. Overall, these results indicate that the HSP system is active in the porcine endometrium during the peri-implantation period, maintaining cellular homeostasis during pregnancy and in conditions of HS.

## Abbreviations

ALT: altrenogest
dpe: days postestrus
HRP: horseradish peroxidase
HS: heat stress
HSP: heat shock proteins
HSR: heat stress response
IgG: Immunoglobulin G
RT: room temperature
SDS: sodium dodecyl sulfate
TGX: Tris–glycine extended
TN: thermal neutral

## References

[CIT0001] Antsiferova, Y. S. 2016. Apoptosis and endometrial receptivity: relationship within vitrofertilization treatment outcome. World J. Obst. Gynecol. 5:87. doi:10.5317/wjog.v5.i1.87

[CIT0002] Asea, A. 2003. Chaperokine-induced signal transduction pathways. Exerc. Immunol. Rev. 9:25–33. PMID: 14686091. 14686091 PMC1822330

[CIT0003] Asea, A. A. A., and A. De Maio. 2007. Heat shock proteins: potent mediators of inflammation and immunity. 1st ed. Dordrecht (The Netherlands): Springer. doi:10.1007/978-1-4020-5585-0

[CIT0004] Auvigne, V., P. Leneveu, C. Jehannin, O. Peltoniemi, and E. Salle. 2010. Seasonal infertility in sows: a five year field study to analyze the relative roles of heat stress and photoperiod. Theriogenology 74:60–66. doi:10.1016/j.theriogenology.2009.12.019 20189636

[CIT0005] Badinga, L., W. W. Thatcher, T. Diaz, M. Drost, and D. Wolfenson. 1993. Effect of environmental heat stress on follicular development and steroidogenesis in lactating Holstein cows. Theriogenology 39:797–810. doi:10.1016/0093-691x(93)90419-6 16727254

[CIT0006] Bagchi, M. K., S. Y. Tsai, M. J. Tsai, and B. W. O’Malley. 1991. Progesterone enhances target gene transcription by receptor free of heat shock proteins hsp90, hsp56, and hsp70. Mol. Cell. Biol. 11:4998–5004. doi:10.1128/mcb.11.10.4998 1922029 PMC361487

[CIT0007] Bai, H., H. Ukita, M. Kawahara, T. Mitani, E. Furukawa, Y. Yanagawa, N. Yabuuchi, H. Kim, and M. Takahashi. 2020. Effect of summer heat stress on gene expression in bovine uterine endometrial tissues. Anim. Sci. J. 91(1):e13474. doi:10.1111/asj.13474 33159383

[CIT0008] Bauersachs, S., and E. Wolf. 2012. Transcriptome analyses of bovine, porcine and equine endometrium during the pre-implantation phase. Anim. Reprod. Sci. 134:84–94. doi:10.1016/j.anireprosci.2012.08.015 22917876

[CIT0009] Baumgard, L. H., R. P. Rhoads, M. L. Rhoads, N. K. Gabler, J. W. Ross, A. F. Keating, R. L. Boddicker, S. Lenka, and V. Sejian. 2012. Impact of climate change on livestock production, environmental stress and amelioration in livestock production. Springer Berlin Heidelberg. p. 413–468. doi:10.1007/978-3-642-29205-7_15

[CIT0010] Bazer, F. W., G. Wu, T. E. Spencer, G. A. Johnson, R. C. Burghardt, and K. Bayless. 2010. Novel pathways for implantation and establishment and maintenance of pregnancy in mammals. Mol. Hum. Reprod. 16:135–152. doi:10.1093/molehr/gap095 19880575 PMC2816171

[CIT0011] Beere, H. M. 2004. The stress of dying’: the role of heat shock proteins in the regulation of apoptosis. J. Cell Sci. 117:2641–2651. doi:10.1242/jcs.01284 15169835

[CIT0012] Bertoldo, M., P. K. Holyoake, G. Evans, and C. G. Grupen. 2010. Oocyte developmental competence is reduced in sows during the seasonal infertility period. Reprod. Fertil. Dev. 22:1222–1229. doi:10.1071/rd10093 20883647

[CIT0013] Bertoldo, M., P. K. Holyoake, G. Evans, and C. G. Grupen. 2011. Follicular progesterone levels decrease during the period of seasonal infertility in sows. Reprod. Domest. Anim. 46:489–494. doi:10.1111/j.1439-0531.2010.01695.x 21083773

[CIT0014] Bidne, K. L., M. J. Dickson, J. W. Ross, L. H. Baumgard, and A. F. Keating. 2018a. Disruption of female reproductive function by endotoxins. Reproduction 155:R169–R181. doi:10.1530/REP-17-0406 29363567

[CIT0015] Bidne, K. L., S. S. Kvidera, J. W. Ross, L. H. Baumgard, and A. F. Keating. 2018b. Impact of repeated lipopolysaccharide administration on ovarian signaling during the follicular phase of the estrous cycle in post-pubertal pigs. J. Anim. Sci. 96:3622–3634. doi:10.1093/jas/sky226 29982469 PMC6127822

[CIT0016] Bidne, K. L., M. R. Romoser, J. W. Ross, L. H. Baumgard, and A. F. Keating. 2019. Heat stress during the luteal phase decreases luteal size but does not affect circulating progesterone in gilts. J. Anim. Sci. 97:4314–4322. doi:10.1093/jas/skz251 31372640 PMC6776266

[CIT0017] Biggs, M. E., K. A. Kroscher, L. D. Zhao, Z. Zhang, E. H. Wall, D. M. Bravo, and R. P. Rhoads. 2020. Dietary supplementation of artificial sweetener and capsicum oleoresin as a strategy to mitigate the negative consequences of heat stress on pig performance. J. Anim. Sci. 98(5):skaa131. doi:10.1093/jas/skaa131 32333770 PMC7233174

[CIT0018] Burel, C., V. Mezger, M. Pinto, M. Rallu, S. Trigon, and M. Morange. 1992. Mammalian heat shock protein families. Expression and functions. Experientia 48:629–634. doi:10.1007/bf02118307 1639170

[CIT0019] Cabezón, F. A., A. P. Schinckel, J. N. Marchant-Forde, J. S. Johnson, and R. M. Stwalley. 2017. Effect of floor cooling on late lactation sows under acute heat stress. Livest. Sci. 206:113–120. doi:10.1016/j.livsci.2017.10.017 PMC6160566 30298134

[CIT0020] Camacho Benítez, A., R. Vasconcellos, P. Lombide, H. Viotti, W. Pérez, N. Cazales, D. Cavestany, G. B. Martin G. Pedrana. 2021. Heat shock protein HSP90 immunoexpression in equine endometrium during oestrus, dioestrus and anoestrus. . Anat. Histol. Embryol. 50:50–57. doi:10.1111/ahe.12598 32776605

[CIT0021] Carra, S., S. Alberti, P. A. Arrigo, J. L. Benesch, I. J. Benjamin, W. C. Boelens, B. Bartelt-Kirbach, B. Brundel, J. Buchner, B. Bukau, et al. 2017. The growing world of small heat shock proteins: from structure to functions. Cell Stress Chaperones 22:601–611. doi:10.1007/s12192-017-0787-8 28364346 PMC5465036

[CIT0022] Carter, F., N. Forde, P. Duffy, M. Wade, T. Fair, M. A. Crowe, A. C. O. Evans, D. A. Kenny, J. F. Roche, and P. Lonergan. 2008. Effect of increasing progesterone concentration from Day 3 of pregnancy on subsequent embryo survival and development in beef heifers. Reprod. Fertility Dev. 20(3):368–375. doi:10.1071/RD07204 18402756

[CIT0023] Chae, J.-I., J. Kim, S. G. Lee, Y.-J. Jeon, D.-W. Kim, Y. Soh, K. S. Seo, H. K. Lee, N.-J. Choi, J. Ryu, et al. 2011. Proteomic analysis of pregnancy-related proteins from pig uterus endometrium during pregnancy. Proteome Sci. 9:41. doi:10.1186/1477-5956-9–41 21791079 PMC3162492

[CIT0024] Cottrell, J. J., F. Liu, A. T. Hung, K. Digiacomo, S. S. Chauhan, B. J. Leury, J. B. Furness, P. Celi, and F. R. Dunshea. 2015. Nutritional strategies to alleviate heat stress in pigs. Anim. Prod. Sci. 55:1391. doi:10.1071/an15255

[CIT0025] Daels, P. F., G. H. Stabenfeldt, J. P. Hughes, K. Odensvik, and H. Kindahl. 1991. Evaluation of progesterone deficiency as a cause of fetal death in mares with experimentally induced endotoxemia. Am. J. Vet. Res. 52:282–288. PMID: 2012339. 2012339

[CIT0026] Dhamad, A. E., Z. Zhou, J. Zhou, and Y. Du. 2016. Systematic proteomic identification of the heat shock proteins (Hsp) that interact with estrogen receptor alpha (ER alpha) and biochemical characterization of the ER alpha-Hsp70 interaction. PLoS One 11:e0160312. doi:10.1371/journal.pone.0160312 27483141 PMC4970746

[CIT0027] Dickson, M. J., K. L. Bidne, B. J. Hale, C. L. Hager, J. T. Seibert, L. H. Baumgard, J. W. Ross, and A. F. Keating. 2016. 1043 Impact of heat stress and metabolic endotoxemia on porcine ovarian function. J. Anim. Sci. 94(Suppl 5):500–500. doi:10.2527/jam2016-104327065120

[CIT0028] Dickson, M. J., C. L. Hager, A. Al-Shaibi, P. Q. Thomas, L. H. Baumgard, J. W. Ross, and A. F. Keating. 2018. Impact of heat stress during the follicular phase on porcine ovarian steroidogenic and phosphatidylinositol-3 signaling. J. Anim. Sci. 96:2162–2174. doi:10.1093/jas/sky144. 29684161 PMC6095433

[CIT0029] Ealy, A. D., M. Drost, and P. J. Hansen. 1993. Developmental changes in embryonic resistance to adverse effects of maternal heat stress in cows. J. Dairy Sci. 76:2899–2905. doi:10.3168/jds.s0022-0302(93)77629-8 8227617

[CIT0030] Evgen’Ev, M. B., D. G. Garbuz, and O. G. Zatsepina. 2014a. Molecular functions of heat shock proteins, heat shock proteins and whole body adaptation to extreme environments. Dordrecht, Heidelberg, New York, London: Springer Netherlands. p. 11–34. doi:10.1007/978-94-017-9235-6_2

[CIT0031] Evgen’Ev, M. B., D. G. Garbuz, and O. G. Zatsepina. 2014b. The discovery of heat shock response system and major groups of heat shock proteins, heat shock proteins and whole body adaptation to extreme environments. Dordrecht, Heidelberg, New York, London: Springer; p. 1–10. doi:10.1007/978-94-017-9235-6_1

[CIT0032] Franczak, A., B. Wojciechowicz, and G. Kotwica. 2013. Transcriptomic analysis of the porcine endometrium during early pregnancy and the estrous cycle. Reprod. Biol. 13:229–237. doi:10.1016/j.repbio.2013.07.001 24011194

[CIT0033] Garrett, J. E., R. D. Geisert, M. T. Zavy, and G. L. Morgan. 1988. Evidence for maternal regulation of early conceptus growth and development in beef cattle. Reproduction 84:437–446. doi:10.1530/jrf.0.0840437 3199361

[CIT0034] Geisert, R. D., G. A. Johnson, and R. C. Burghardt. 2015. Implantation and establishment of pregnancy in the pig. Switzerland: Springer International Publishing; p. 137–163. doi:10.1007/978-3-319-15856-3_8 26450498

[CIT0035] Gross, T. S., D. J. Putney, F. W. Bazer, and W. W. Thatcher. 1989. Effect of in-vitro heat stress on prostaglandin and protein secretion by endometrium from pregnant and cyclic gilts at Day 14 after oestrus. Reproduction 85:541–550. doi:10.1530/jrf.0.0850541 2703994

[CIT0036] Hale, B. J., C. L. Hager, J. T. Seibert, J. T. Selsby, L. H. Baumgard, A. F. Keating, and J. W. Ross. 2017. Heat stress induces autophagy in pig ovaries during follicular development. Biol. Reprod. 97:426–437. doi:10.1093/biolre/iox097 29025092

[CIT0037] Hansen, P. J. 2009. Effects of heat stress on mammalian reproduction. Philos. Trans. R. Soc. Lond. B Biol. Sci. 364:3341–3350. doi:10.1098/rstb.2009.0131 19833646 PMC2781849

[CIT0038] Jaiswal, M. K., V. Agrawal, and Y. K. Jaiswal. 2013. Lipopolysaccharide drives alternation of heat shock proteins and induces failure of blastocyst implantation in mouse. Biol. Reprod. 88:162. doi:10.1095/biolreprod.113.108068 23677983

[CIT0039] Jee, B., R. Dhar, S. Singh, and S. Karmakar. 2021. Heat Shock Proteins and Their Role in Pregnancy: Redefining the Function of “Old Rum in a New Bottle”. Front. Cell Dev. Biol. 9:648463. doi:10.3389/fcell.2021.648463 33996811 PMC8116900

[CIT0040] Jin, Y. X., J. Y. Lee, S. H. Choi, T. Kim, X. S. Cui, and N. H. Kim. 2007. Heat shock induces apoptosis related gene expression and apoptosis in porcine parthenotes developing in vitro. Anim. Reprod. Sci. 100:118–127. doi:10.1016/j.anireprosci.2006.06.017 16919406

[CIT0041] Kraeling, R. R., and S. K. Webel. 2015. Current strategies for reproductive management of gilts and sows in North America. J. Anim. Sci. Biotechnol. 6:1–14. doi:10.1186/2049-1891-6-3 25838898 PMC4382856

[CIT0042] van Leeuwen, J. J., M. R. Martens, J. Jourquin, M. A. Driancourt, B. Kemp, and N. M. Soede. 2011. Effects of altrenogest treatments before and after weaning on follicular development, farrowing rate, and litter size in sows. J. Anim. Sci. 89:2397–2406. doi:10.2527/jas.2010-3752 21421833

[CIT0043] Li, H., S. Guo, L. Cai, W. Ma, and Z. Shi. 2017. Lipopolysaccharide and heat stress impair the estradiol biosynthesis in granulosa cells via increase of HSP70 and inhibition of smad3 phosphorylation and nuclear translocation. Cell. Signal. 30:130–141. doi:10.1016/j.cellsig.2016.12.004 27940052

[CIT0044] Lonergan, P., N. Forde, and T. Spencer. 2016. Role of progesterone in embryo development in cattle. Reprod. Fertil. Dev. 28:66–74. doi:10.1071/rd15326 27062875

[CIT0045] De Maio, A. 2011. Extracellular heat shock proteins, cellular export vesicles, and the Stress Observation System: a form of communication during injury, infection, and cell damage. Cell Stress Chaperones 16:235–249. doi:10.1007/s12192-010-0236-4 20963644 PMC3077223

[CIT0046] De Maio, A., and D. Vazquez. 2013. Extracellular heat shock proteins: a new location, a new function. Shock (Augusta, Ga.) 40:239–246. doi:10.1097/SHK.0b013e3182a185ab PMC4351735 23807250

[CIT0047] Martinat-Botte, F., F. Bariteau, Y. Forgerit, C. Macar, P. Poirier, and M. Terqui. 1995. Synchronization of oestrus in gilts with altrenogest: effects on ovulation rate and foetal survival. Anim. Reprod. Sci. 39:267–274. doi:10.1016/0378-4320(95)01396-H

[CIT0048] Mathew, D. J., M. C. Lucy, and G. R. D. 2016. Interleukins, interferons, and establishment of pregnancy in pigs. Reproduction 151:R111–R122. doi:10.1530/REP-16-0047 27001998

[CIT0049] Mayorga, E. J., J. W. Ross, A. F. Keating, R. P. Rhoads, and L. H. Baumgard. 2020. Biology of heat stress; the nexus between intestinal hyperpermeability and swine reproduction. Theriogenology 154:73–83. doi:10.1016/j.theriogenology.2020.05.023 32531658

[CIT0050] Muro, B. B. D., R. F. Carnevale, D. F. Leal, M. A. Torres, M. V. Mendonça, D. H. Nakasone, C. H. G. Martinez, G. M. Ravagnani, M. S. Monteiro, A. P. Poor, et al. 2020. Supplemental progesterone during early pregnancy exerts divergent responses on embryonic characteristics in sows and gilts. Animal 14:1234–1240. doi:10.1017/s1751731119002982 31907084

[CIT0051] Muro, B. B. D., D. F. Leal, R. F. Carnevale, M. A. Torres, M. V. Mendonça, D. H. Nakasone, C. H. G. Martinez, G. M. Ravagnani, M. S. Monteiro, A. P. Poor, et al. 2021. Altrenogest during early pregnancy modulates uterine glandular epithelium and endometrial growth factor expression at the time implantation in pigs. Anim. Reprod. 18. doi:10.1590/1984-3143-ar2020-0431 PMC8189350 34122654

[CIT0052] Neuer, A. 2000. The role of heat shock proteins in reproduction. Hum. Reprod. Update 6:149–159. doi:10.1093/humupd/6.2.149 10782573

[CIT0053] Neuer, A., S. D. Spandorfer, P. Giraldo, S. Dieterle, Z. Rosenwaks, and S. S. Witkin. 2000. The role of heat shock proteins in reproduction. Hum. Reprod. Update 6:149–159. doi:10.1093/humupd/6.2.149 10782573

[CIT0054] Nteeba, J., M. V. Sanz-Fernandez, R. P. Rhoads, L. H. Baumgard, J. W. Ross, and A. F. Keating. 2015. Heat stress alters ovarian insulin-mediated phosphatidylinositol-3 kinase and steroidogenic signaling in gilt ovaries. Biol. Reprod. 92:148. doi:10.1095/biolreprod.114.126714 25926439

[CIT0055] Omtvedt, I. T., R. E. Nelson, L. E. Ronnie, D. F. Stephens, and E. J. Turman. 1971. Influence of heat stress during early, mid and late pregnancy of gilts. J. Anim. Sci. 32(2):312–317. doi:10.2527/jas1971.322312x 5543028

[CIT0056] Padwick, M. L., M. Whitehead, and R. J. B. King. 1994. Hormonal regulation of HSP27 expression in human endometrial epithelial and stromal cells. Mol. Cell. Endocrinol. 102:9–14. doi:10.1016/0303-7207(94)90091-4 7926277

[CIT0057] Pennarossa, G., S. Maffei, M. M. Rahman, G. Berruti, T. A. Brevini, and F. Gandolfi. 2012. Characterization of the constitutive pig ovary heat shock chaperone machinery and its response to acute thermal stress or to seasonal variations. Biol. Reprod. 87:119. doi:10.1095/biolreprod.112.104018 23018186

[CIT0058] De Rensis, F., A. J. Ziecik, and R. N. Kirkwood. 2017. Seasonal infertility in gilts and sows: aetiology, clinical implications and treatments. Theriogenology 96:111–117. doi:10.1016/j.theriogenology.2017.04.004 28532826

[CIT0059] Richter, K., M. Haslbeck, and J. Buchner. 2010. The heat shock response: life on the verge of death. Mol. Cell 40:253–266. doi:10.1016/j.molcel.2010.10.006 20965420

[CIT0060] Romoser, M. R., K. L. Bidne, L. H. Baumgard, A. F. Keating, and J. W. Ross. 2022. Effects of increased ambient temperature and supplemental altrenogest prior to pregnancy establishment in gilts. J. Anim. Sci. 100(2):skac007. doi:10.1093/jas/skac007 35018454 PMC8865011

[CIT0061] Ross, J. W., M. D. Ashworth, A. G. Hurst, J. R. Malayer, and R. D. Geisert. 2003a. Analysis and characterization of differential gene expression during rapid trophoblastic elongation in the pig using suppression subtractive hybridization. Reprod. Biol. Endocrinol. 1:23. doi:10.1186/1477-7827-1-23 12646053 PMC151795

[CIT0062] Ross, J. W., M. D. Ashworth, D. R. Stein, O. P. Couture, C. K. Tuggle, and R. D. Geisert. 2009. Identification of differential gene expression during porcine conceptus rapid trophoblastic elongation and attachment to uterine luminal epithelium. Physiol. Genom. 36(3):140–148. doi:10.1152/physiolgenomics.00022.2008 19033546

[CIT0063] Ross, J. W., B. J. Hale, J. T. Seibert, M. R. Romoser, M. K. Adur, A. F. Keating, and L. H. Baumgard. 2017. Physiological mechanisms through which heat stress compromises reproduction in pigs. Mol. Reprod. Dev. 84:934–945. doi:10.1002/mrd.22859 28667793

[CIT0064] Ross, J. W., J. R. Malayer, J. W. Ritchey, and R. D. Geisert. 2003b. Characterization of the interleukin-1beta system during porcine trophoblastic elongation and early placental attachment. Biol. Reprod. 69:1251–1259. doi:10.1095/biolreprod.103.015842 12801990

[CIT0065] Roth, Z., and D. Wolfenson. 2016. Comparing the effects of heat stress and mastitis on ovarian function in lactating cows: basic and applied aspects. Domest Anim. Endocrinol. 56:S218–S227. doi:10.1016/j.domaniend.2016.02.013 27345320

[CIT0066] Satterfield, M. C., F. W. Bazer, and T. E. Spencer. 2006. Progesterone regulation of preimplantation conceptus growth and galectin 15 (LGALS15) in the ovine uterus. Biol. Reprod. 75(2):289–296. doi:10.1095/biolreprod.106.052944 16707766

[CIT0067] Seibert, J. T., M. K. Adur, R. B. Schultz, P. Q. Thomas, Z. E. Kiefer, A. F. Keating, L. H. Baumgard, and J. W. Ross. 2019. Differentiating between the effects of heat stress and lipopolysaccharide on the porcine ovarian heat shock protein response. J. Anim. Sci. 97:4965–4973. doi:10.1093/jas/skz343 31782954 PMC6915215

[CIT0068] Shah, M., J. Stanek, and S. Handwerger. 1998. Differential localization of heat shock proteins 90, 70, 60 and 27 in human decidua and placenta during pregnancy. Histochem. J. 30:509–518. doi:10.1023/a:1003259907014 10192534

[CIT0069] Spencer, T. E., N. Forde, and P. Lonergan. 2016. The role of progesterone and conceptus-derived factors in uterine biology during early pregnancy in ruminants. J. Dairy Sci. 99:5941–5950. doi:10.3168/jds.2015-10070 26387021

[CIT0070] St-Pierre, N. R., B. Cobanov, and G. Schnitkey. 2003. Economic losses from heat stress by US livestock industries. J. Dairy Sci. 86:E52–E77. doi:10.3168/jds.s0022-0302(03)74040-5

[CIT0071] Sørensen, J. G., T. N. Kristensen, and V. Loeschcke. 2003. The evolutionary and ecological role of heat shock proteins. Ecol. Lett. 6:1025–1037. doi:10.1046/j.1461-0248.2003.00528.x

[CIT0072] Tabibzadeh, S., Q. F. Kong, P. G. Satyaswaroop, and A. Babaknia. 1996. Heat shock proteins in human endometrium throughoutthe menstrual cycle. Hum. Reprod. 11:633–640. doi:10.1093/humrep/11.3.633 8671282

[CIT0073] Tast, A., O. A. T. Peltoniemi, J. V. Virolainen, and R. J. Love. 2002. Early disruption of pregnancy as a manifestation of seasonal infertility in pigs. Anim. Reprod. Sci. 74:75–86. doi:10.1016/s0378-4320(02)00167-7 12379377

[CIT0074] de Thonel, A., A. Le Mouel, and V. Mezger. 2012. Transcriptional regulation of small HSP-HSF1 and beyond. Int. J. Biochem. Cell Biol. 44:1593–1612. doi:10.1016/j.biocel.2012.06.012 22750029

[CIT0075] Tompkins, E. C., C. J. Heidenreich, and M. Stob. 1967. Effect of post-breeding thermal stress on embyronic mortality in swine. J. Anim. Sci. 26:377–380. doi:10.2527/jas1967.262377x

[CIT0076] van Wettere, W. H. E. J., K. L. Kind, K. L. Gatford, A. M. Swinbourne, S. T. Leu, P. T. Hayman, J. M. Kelly, A. C. Weaver, D. O. Kleemann, and S. K. Walker. 2021. Review of the impact of heat stress on reproductive performance of sheep. J. Anim. Sci. Biotechnol. 12(1). doi:10.1186/s40104-020-00537-z PMC7883430 33583422

[CIT0077] Wang, Z., B. S. Liu, X. Y. Wang, Q. H. Wei, H. Tian, and L. Q. Wang. 2018. Effects of altrenogest on reproductive performance of gilts and sows: A meta-analysis. Anim. Reprod. Sci. 197:10–21. doi:10.1016/j.anireprosci.2018.08.035 30197055

[CIT0078] Welch, W. J., M. j. Gething, A. R. Clarke, P. Viitanen, P. Lund, I. G. Haas, C. Georgopoulos, R. J. Ellis, R. A. Laskey, and G. H. Lorimer. 1993. Heat shock proteins functioning as molecular chaperones: their roles in normal and stressed cells. Philos. Trans. R. Soc. Lond. B Biol. Sci. 339(1289):327–333. doi:10.1098/rstb.1993.0031 8098537

[CIT0079] Witkin, S. S., and I. M. Linhares. 2010. Heat shock proteins and fertility, heat shock proteins and whole body physiology. Dordrecht, Heidelberg, London, New York: Springer; p. 151–162. doi:10.1007/978-90-481-3381-9_9

[CIT0080] Yu, C., S. V. Tracy, P. L. Peter, and G. N. a. R. W. C. Earl. 2007. Heat shock paradox and a new role of heat shock proteins and their receptors as anti-inflammation targets. Inflamm. Allergy Drug Targets 6(2):91–100. doi:10.2174/187152807780832274 17692032

[CIT0081] Yuan, J. X., L. J. Xiao, C. L. Lu, X. S. Zhang, T. Liu, M. Chen, Z. Y. Hu, F. Gao, and Y. X. Liu. 2009. Increased expression of heat shock protein 105 in rat uterus of early pregnancy and its significance in embryo implantation. Reprod. Biol. Endocrinol. 7:23. doi:10.1186/1477-7827-7-23 19284651 PMC2667524

